# Postsynaptic density protein 95 (PSD-95) is transported by KIF5 to dendritic regions

**DOI:** 10.1186/s13041-019-0520-x

**Published:** 2019-11-21

**Authors:** Ki-Seo Yoo, Kina Lee, Jun-Young Oh, Hyoeun Lee, Hyungju Park, Young Seok Park, Hyong Kyu Kim

**Affiliations:** 1 Department of Medicine and Microbiology, Graduate Program in Neuroscience, College of Medicine, Chungbuk National University, 1 Chungdae-ro, Seowon-gu, Cheongju, 28644 South Korea; 2 Department of Structure and Function of Neural Network, Korea Brain Research Institute, 61 Cheomdan-ro, Dong-gu, Daegu, 41068 South Korea; 3 Department of Neurosurgery, Graduate Program in Neuroscience, College of Medicine, Chungbuk National University, 1 Chungdae-ro, Seowon-gu, Cheongju, 28644 South Korea

**Keywords:** PSD-95, KIF5, Glutamate receptor 1, Dendritic transport

## Abstract

Postsynaptic density protein 95 (PSD-95) is a pivotal postsynaptic scaffolding protein in excitatory neurons. Although the transport and regulation of PSD-95 in synaptic regions is well understood, dendritic transport of PSD-95 before synaptic localization still remains to be clarified. To evaluate the role of KIF5, conventional kinesin, in the dendritic transport of PSD-95 protein, we expressed a transport defective form of KIF5A (ΔMD) that does not contain the N-terminal motor domain. Expression of ΔMD significantly decreased PSD-95 level in the dendrites. Consistently, KIF5 was associated with PSD-95 in in vitro and in vivo assays. This interaction was mediated by the C-terminal tail regions of KIF5A and the third PDZ domain of PSD-95. Additionally, the ADPDZ3 (the association domain of NMDA receptor and PDZ3 domain) expression significantly reduced the levels of PSD-95, glutamate receptor 1 (GluA1) in dendrites. The association between PSD-95 and KIF5A was dose-dependent on Staufen protein, suggesting that the Staufen plays a role as a regulatory role in the association. Taken together, our data suggest a new mechanism for dendritic transport of the AMPA receptor-PSD-95.

## Introduction

In excitatory neurons, neuronal excitability changes synaptic function [1–5] by regulating expression level of synaptic scaffold proteins such as postsynaptic density protein 95 (PSD-95)—a member of the membrane-associated guanylate kinase (MAGUK) class of proteins at synapses [6–8]. PSD-95 regulates the trafficking and localization of glutamate receptors such as α-amino-3-hydroxy-5-methyl-4-isoxazolepropionic acid (AMPA)-type or *N*-methyl-D-aspartate (NMDA) type-receptors [4]. Overexpression of PSD-95 enhances the amplitude of the AMPA receptor-mediated synaptic current [1, 9, 10]. For all these reasons, PSD-95 has been implicated in synaptic development, plasticity [11, 12], and defects across several disorders [13, 14]. Despite essential roles of PSD-95 in synaptic functions, its transport and localization to dendrites are partially understood [15]. One study reported that kinesin superfamily protein 1Bα (KIF1Bα)—a member of the kinesin-3 family—associates with MAGUKs such as PSD-95 and synapse-associated protein (SAP)-97, as well as synaptic scaffolding molecule (S-SCAM) and membrane associated guanylate kinase inverted-2 (MAGI-2), suggesting that KIF1Bα functions as a motor protein for synaptic localization of these proteins [15].

KIF proteins transport a various molecules, including proteins, synaptic vesicles, and mitochondria along the microtubules cytoskeleton of an axon or dendrites to synaptic regions [16]. In particular, KIF5, which belongs to the recently classified kinesin-1 family, consists of three isoforms (A, B and C) [17]. It transports ribonucleoprotein complexes, synaptic vesicles, mitochondria, AMPA receptor vesicles, tyrosine receptor kinase B (TrkB)-containing vesicles [18], and γ-aminobutyric acid (GABA_A_) receptor vesicles [19] in neurons, as well as slowly transported cargo proteins in axons [20]. KIF5 localizes AMPA receptor vesicles to the postsynaptic regions interacting with glutamate receptor interacting protein 1 (GRIP1)—a scaffolding protein similar to a MAGUK and a member of the PSD-95/SAP90/discs large homology (DLG)/zona occludens (ZO)-1 (PDZ)-domain proteins, functioning as a synaptic scaffolding protein [21, 22]. Several studies have reported that MAGUKs are transported to membrane regions by KIF13B [23, 24].

In the present study, we revealed that KIF5 as a motor protein involved in PSD-95 dendritic transport. The C-terminal tail region of KIF5A was associated with the PDZ3 domain of PSD-95. The expression of the ADPDZ3 domain, which includes an NMDA receptor-associated domain (AD), significantly decreased levels of PSD-95 and surface glutamate receptor 1 (GluA1) at the postsynaptic site. Finally, we found that the KIF5A-PSD-95 complex colocalized with GluA1-immuopositive particles in dendritic regions, indicating that KIF5A mediates the transport of both PSD-95 and GluA1-containing vesicles. Thus, we suggest that PSD-95 works as both a scaffolding protein in the excitatory synapses and an adaptor between a cargo and motor proteins.

## Results

### Expression of a dominant-negative form of KIF5A reduces level of PSD-95 in dendrites

**Fig. 1:**
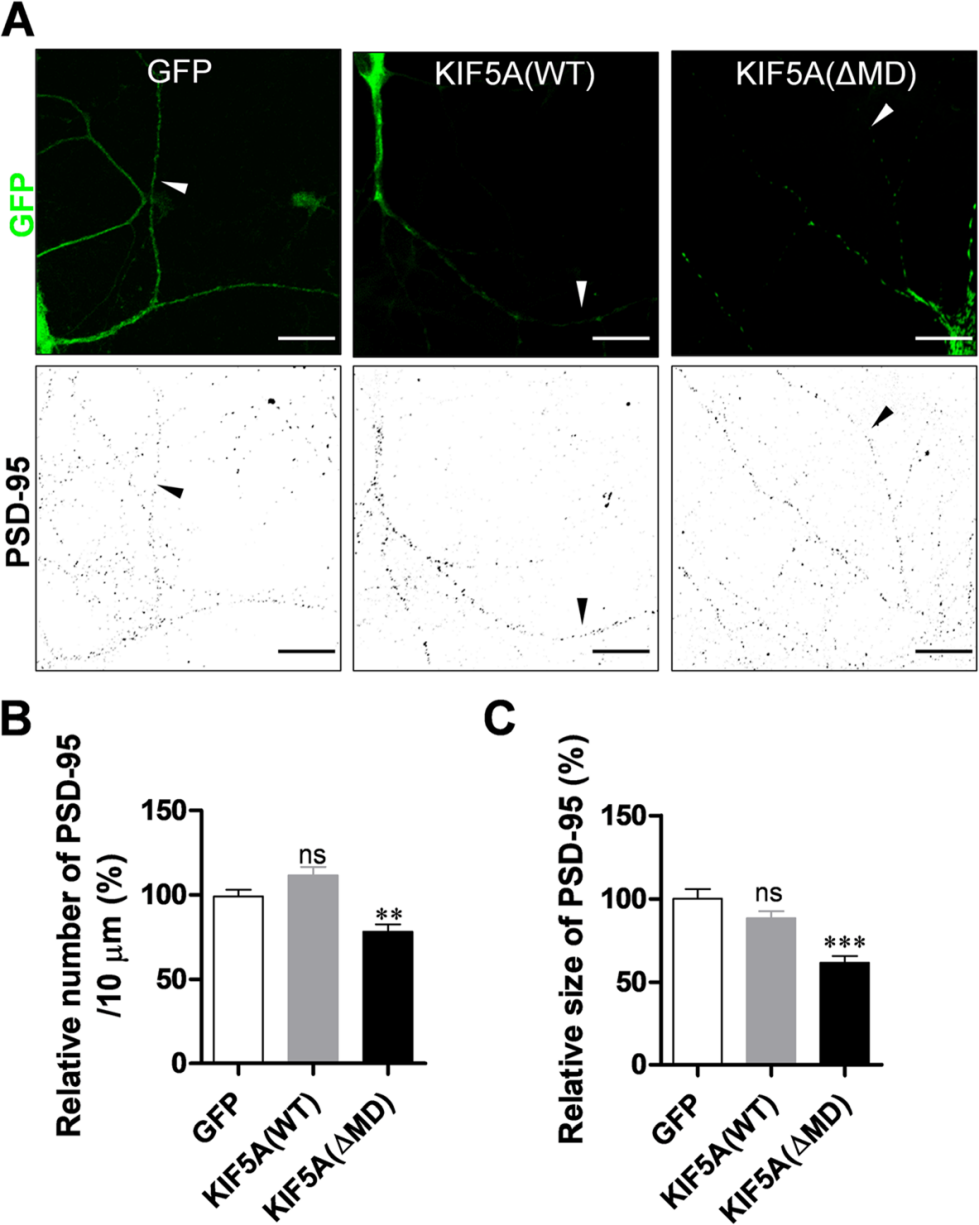
Expression of KIF5A ΔMD mutant reduces the number and average size of PSD-95 particles in dendrites. Cultured rat hippocampal neurons were infected with Sindbis viruses encoding GFP, GFP-KIF5A (WT), or GFP-KIF5A (ΔMD), incubated for 9 h, and immunostained with monoclonal anti-PSD-95 antibody. **a** Representative images of infected neurons. Scale bar: 20 μm. **b** ΔMD expression significantly reduces the number of PSD-95 particles (GFP: 100.00% ± 3.88%, *n* = 31 dendrites, 3417 μm; KIF5A [WT]: 111.70% ± 4.91%, n = 31 dendrites, 3724 μm; KIF5A [ΔMD]: 78.48% ± 4.23%, *n* = 29 dendrites, 3287 μm; Kruskal–Wallis test: *P* < 0.0001; Dunn’s multiple comparison test: ** *P* < 0.01, ns: not significant). **c** ΔMD expression significantly reduces the average size of PSD-95 particles (GFP: 100.00% ± 5.83%, n = 31 dendrites; KIF5A [WT]: 88.39% ± 4.27%, n = 31 dendrites; KIF5A [ΔMD]: 61.71% ± 4.00%, n = 29 dendrites; Kruskal–Wallis test: *P* < 0.0001; Dunn’s multiple comparison test: *** *P* < 0.001, ns: not significant). N values denote the dendritic number from n neurons

To examine the relevance of kinesin motor protein to dendritic transport of PSD-95, we expressed either green fluorescent protein (GFP), GFP-tagged wild type (WT) KIF5A (which is enriched in neurons) [17], or a dominant-negative mutant of KIF5A lacking the N-terminal motor domain (ΔMD) [25] in cultured hippocampal neurons. We then examined PSD-95 particles in dendrites. Consistent with our previous results [26], ΔMD expression significantly reduced the number and average size of PSD-95 particles by 78.48 and 61.71% (Fig. 1), respectively, indicating that inhibition of KIF5A functions reduces PSD-95 levels in dendrites. Interestingly, expression of the WT did not induce any significant change.

### PSD-95 colocalizes with KIF5

**Fig. 2:**
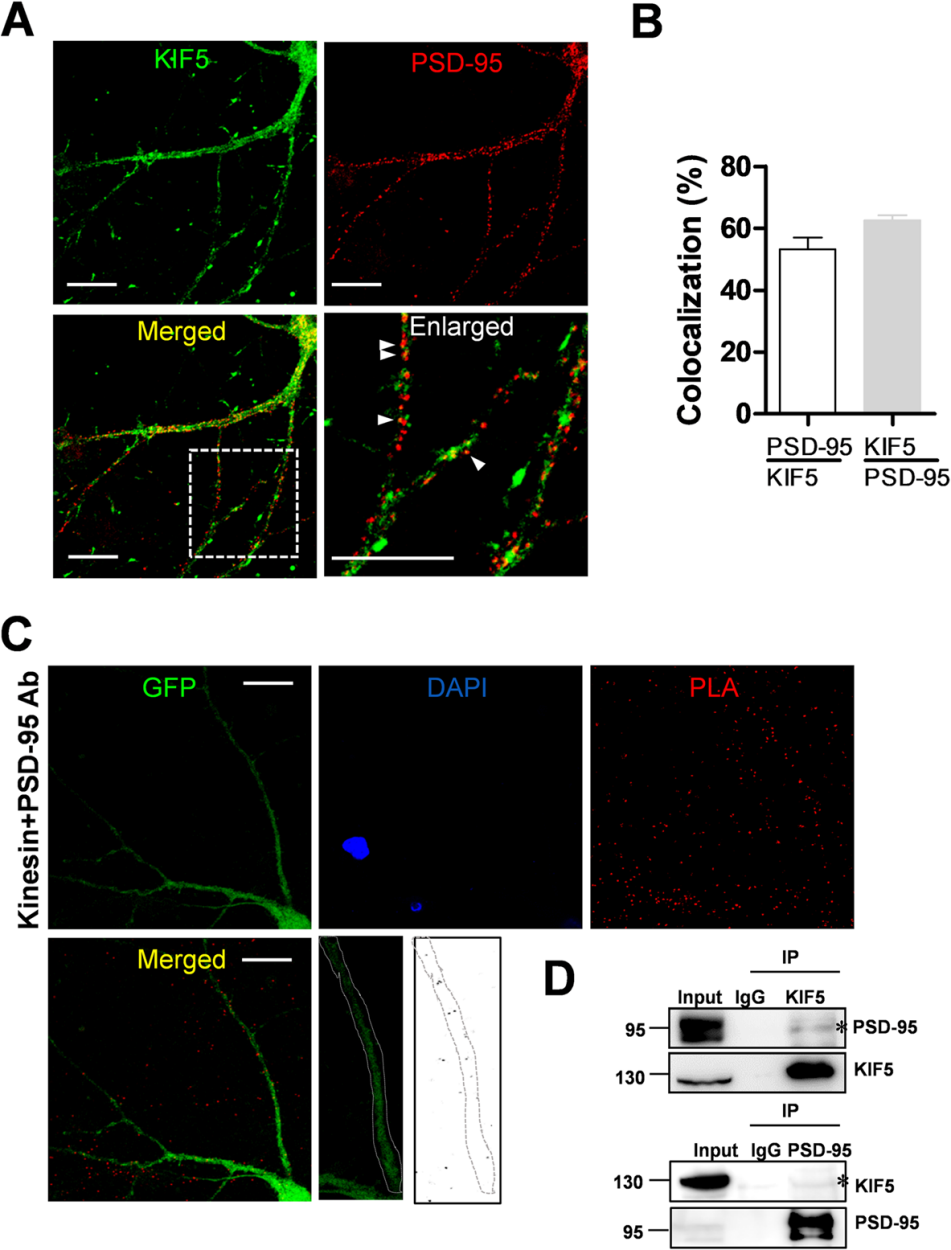
PSD-95 is colocalized with KIF5. **a** Representative images of immunostaining. Scale bar: 20 μm. Cultured neurons were immunostained with polyclonal anti-PSD-95 antibody and monoclonal anti-KIF5 antibody (H2), followed by Alexa Fluor® 488 anti-rabbit IgG antibody for PSD-95 and Cy3-conjugated anti-mouse IgG antibody for KIF5. **b** Results of co-localization analysis. 53.17% ± 3.86% (*n* = 19 dendrites, 1354 μm) of KIF5-immunopositve puncta are colocalized with PSD-95-immunopositve puncta and 62.55% ± 1.69% (n = 19 dendrites, 1354 μm) of PSD-95-immunopositive puncta are colocalized with KIF5-immunopositve puncta. **c** Results of proximity ligation assay. Cultured neurons were infected with Sindbis viruses encoding GFP and subjected to PLA. Red dots in PLA indicate an interaction between the two proteins. Scale bar: 20 μm. **d** Results of IP assays using rat brain lysates. In total 500 μg of rat brain lysate was used in the IP assays using 3 μg monoclonal anti-KIF5 antibodies or polyclonal PSD-95 antibodies. These were analyzed by Western blotting using the antibody indicated. Asterisks indicate interaction bands in the Western blot assays

In order to specify which isoform of KIF5 interacts with the PSD-95, we examined an association between isoform of KIF5 and PSD-95. The results showed that the PSD-95 interacts with all isoforms of KIF5s (Additional file 1: Figure S1). We examined the localization of PSD-95 and KIF5 in cultured neurons. As shown in Fig. 2a and b, a significant number of puncta immunopositive for KIF5 were colocalized with puncta of PSD-95 (PSD-95/KIF5: 53.17% ± 3.86%) and many puncta immunopositive for PSD-95 were colocalized with puncta of KIF5 (KIF5/PSD-95: 62.55% ± 1.69%). To further visualize the colocalization of two proteins in vivo, we performed a proximity ligation assay (PLA), which also indicated an association between the two proteins. The results of PLA showed that endogenous KIF5 and PSD-95 interacts in dendrites (Fig. 2c). Supporting this result, our immunoprecipitation analysis using anti-KIF5 or anti-PSD-95 antibody and rat brain lysates also showed interaction between endogenous KIF5 and PSD-95 (Fig. 2d). Taken together, these results show that PSD-95 may interact with KIF5 in neurons. Although all KIF5 members had showed the interaction with PSD-95 (Additional file 1: Figure S1), we decided to focus on the role of KIF5A, having pan-neuronal expression pattern [17], to study detailed mechanisms of the interaction and dendritic transport.

### Interaction between PSD-95 and KIF5A requires the PDZ3 domain of PSD-95

**Fig. 3:**
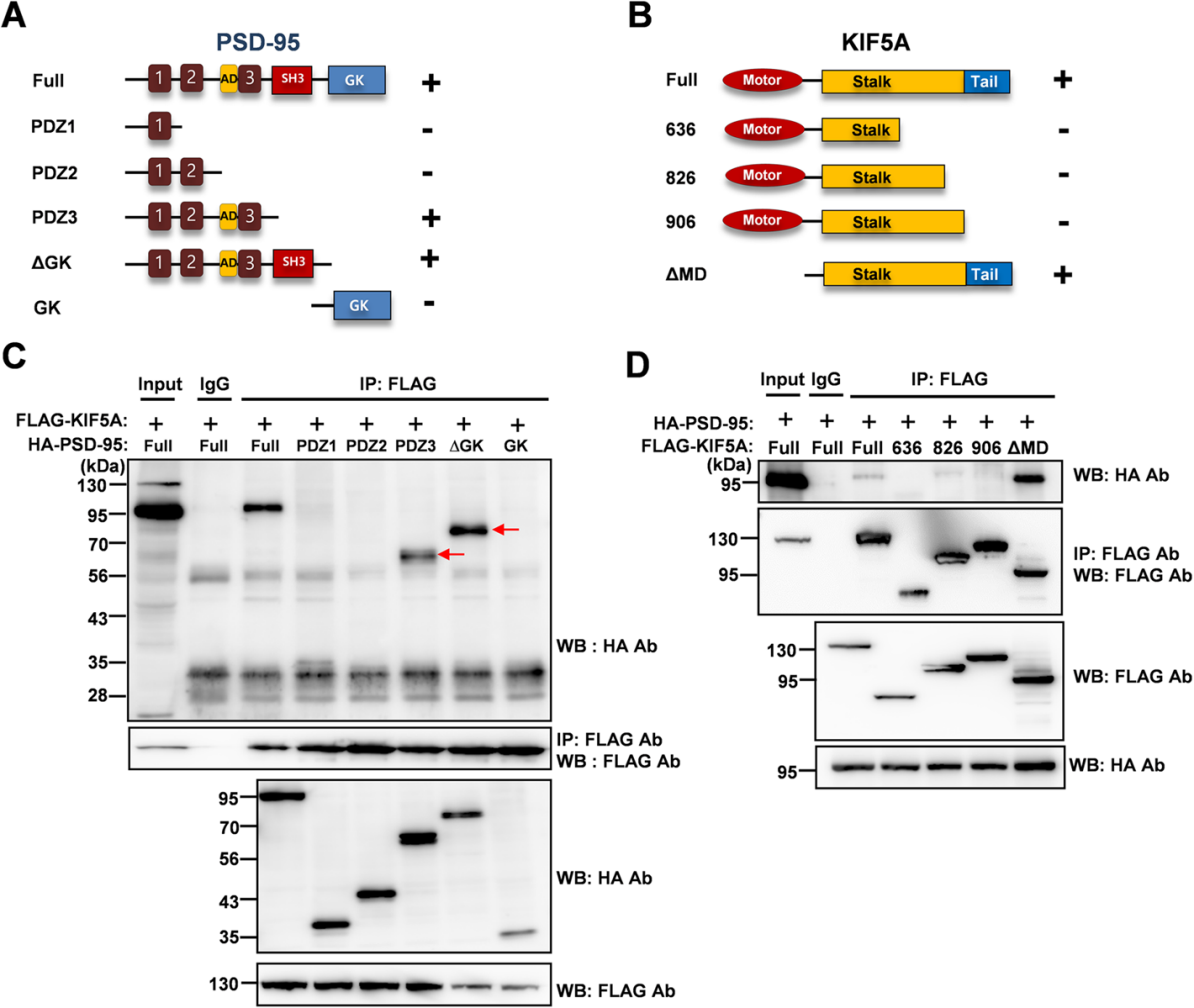
The PDZ3 domain of PSD-95 and the tail region of KIF5A are required for the interaction. Full-length PSD-95 or truncated mutants with full-length KIF5A (**a**, **c**), and full-length KIF5A or truncated mutants with full-length PSD-95 (**b**, **d**) were transfected into HEK 293T cells. The cell lysates were then used in immunoprecipitation assays. **a** Truncated mutants of PSD-95 and interaction with full-length KIF5A, analyzed by co-IP assays. GK: Guanosine monophosphate kinase. **b** Truncated mutants of KIF5A and interaction with full-length PSD-95, analyzed by co-IP assays. **c**, **d** Results of co-IP assays. The lysates were precipitated with anti-FLAG antibody, and the immunoprecipitates were analyzed in Western blotting assays using anti-HA antibody

**Fig. 4:**
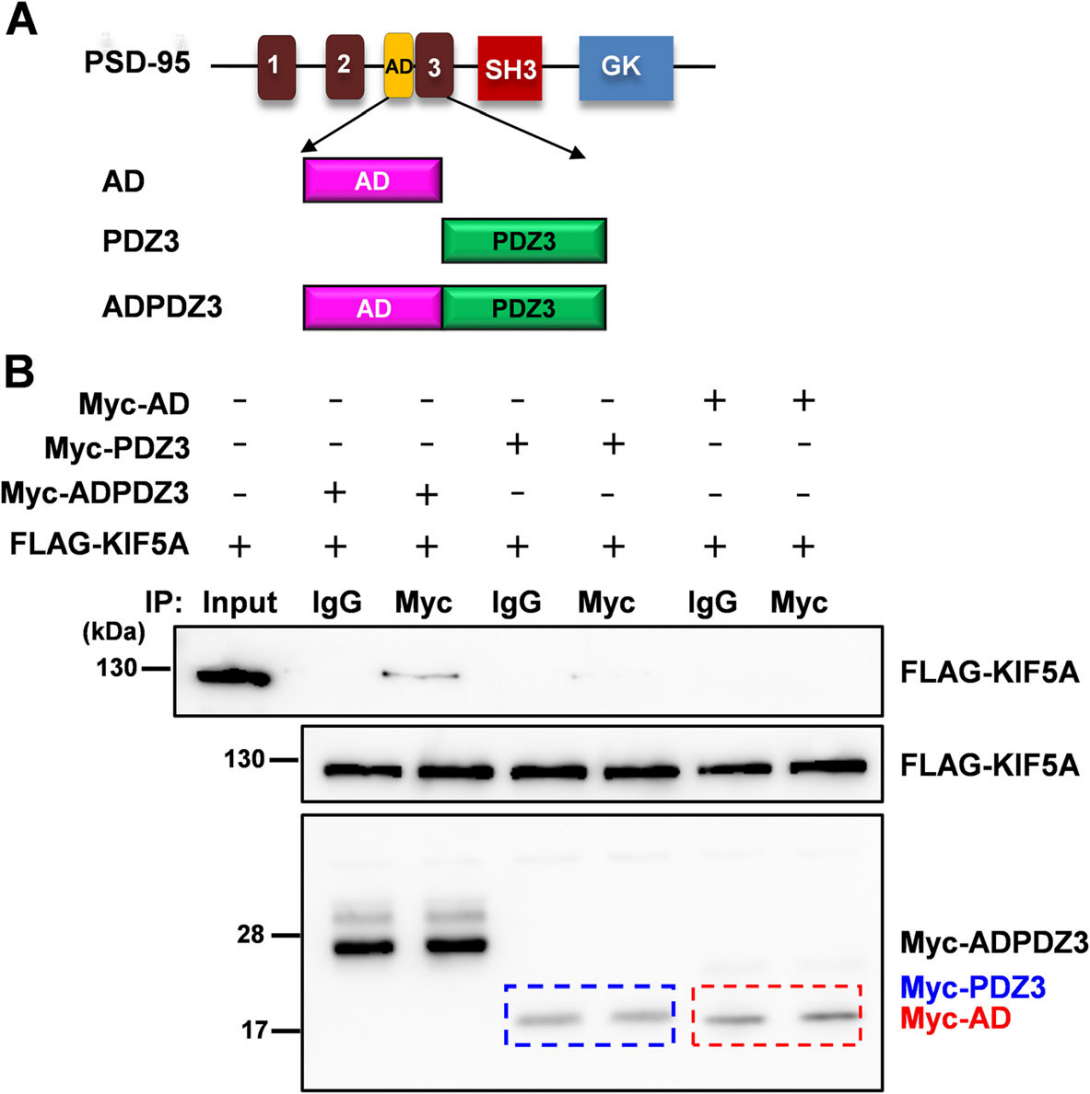
ADPDZ3 domain is required for the interaction. To identify which domains are necessary for the interaction, AD (PDZ-associated domain of the NMDA receptor), PDZ3, ADPDZ3 were constructed and used in co-IP assays with full-length KIF5A. **a** Truncated mutants of PSD-95. **b** Results of co-IP assays. The blue dotted box indicates the expression of Myc-tagged PDZ3, while the red one indicate Myc-tagged AD

In order to identify which domains of PSD-95 and KIF5A are required for this interaction, we constructed a series of truncated mutants of PSD-95 and KIF5A. First, we examined whether FLAG-tagged full-length KIF5A could interact with hemagglutinin (HA)-tagged truncated PSD-95 mutants. As shown in Fig. 3a and c, the third PDZ domain (PDZ3) was required for interaction with KIF5A. Next, we examined whether HA-tagged full-length PSD-95 could interact with FLAG-tagged truncated KIF5A mutants. Consistent with previous reports [27, 28], the results indicated that the tail region of KIF5A was required for the interaction (Fig. 3d). To identify the necessary regions more closely, we constructed plasmids to express either the PDZ-associated domain of NMDA receptors (AD) or PDZ3 and examined the interaction with full-length KIF5A. The PDZ3 domain was sufficient for the interaction with KIF5A, although this interaction was weaker than that with ADPDZ3 (Fig. 4). Thus, in the following experiments, we used ADPDZ3 to block the interaction between the two proteins. Our data indicate that the tail region of KIF5A and PDZ3 domain of PSD-95 are required for the interaction between KIF5A and PSD-95.

### ADPDZ3 expression reduces PSD-95 level in dendrites

**Fig. 5:**
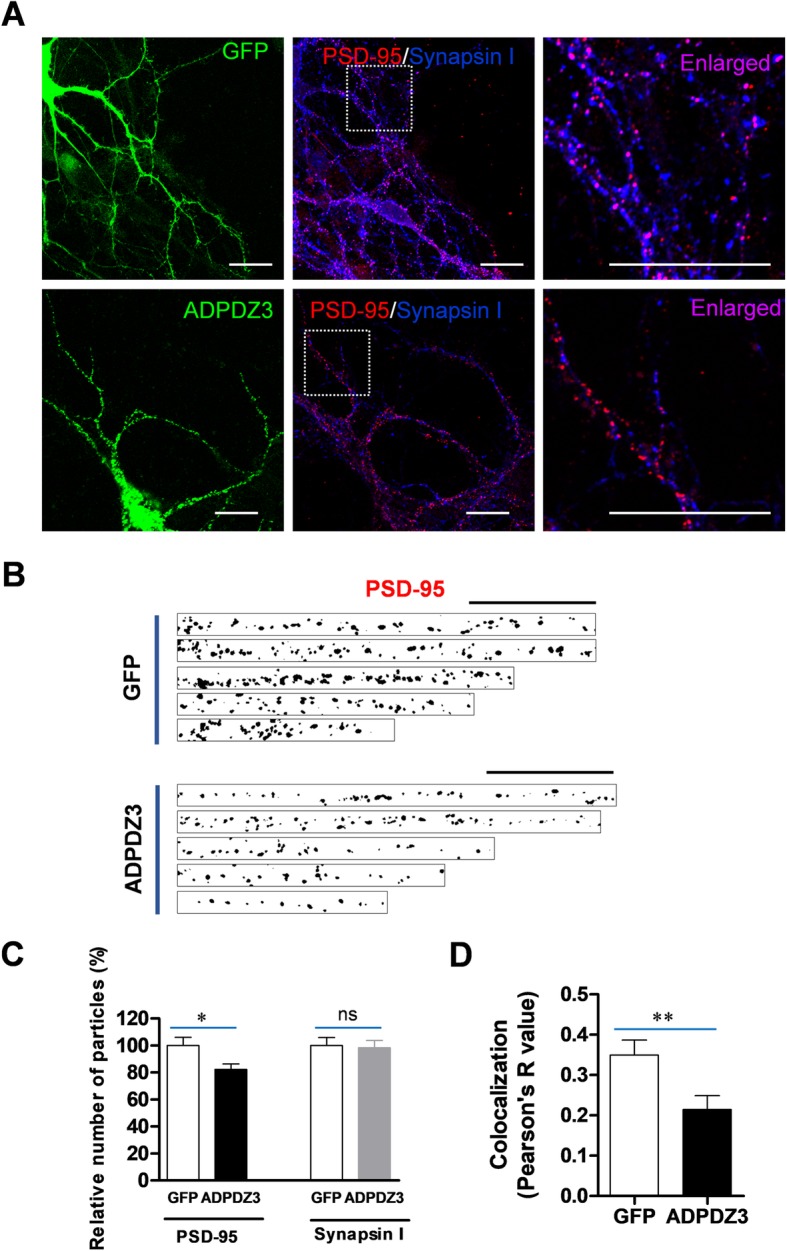
ADPDZ3 expression reduces the level of PSD-95 in dendrites. Cultured rat hippocampal neurons were infected with Sindbis viruses encoding GFP or GFP-ADPDZ3 and incubated for 9 h to allow expression. The cultures were then subjected to immunostaining using monoclonal anti-PSD-antibody and Cy3-conjugated anti-mouse IgG antibody or polyclonal anti-synapsin I antibody and Alexa Fluor® 647 anti-rabbit IgG antibody. They were then visualized using confocal microscopy. **a** Representative images of expressed neurons. Arrows indicate analyzed dendrites. Scale bar: 20 μm. **b**, **c** ADPDZ3 expression reduced the number of PSD-95 particles in the dendrites (GFP: 100.00% ± 6.08%, *n* = 32, 1766 μm; ADPDZ3: 82.27% ± 4.03%, *n* = 35, 1973 μm; * *P* < 0.05), but not that of synapsin I particles (GFP: 100.00% ± 6.00%, n = 32, 1766 μm; ADPDZ3: 98.36% ± 5.41%, n = 35, 1973 μm; ns: not significant). Scale bar: 20 μm. **d** ADPDZ3 expression significantly reduced colocalization between PSD-95 and synapsin I (GFP: 0.350 ± 0.037, *n* = 32; ADPDZ3: 0.214 ± 0.035, *n* = 37; ** *P* < 0.01). N values indicate number of dendrites from multiple neurons derived from three different batches

Because ADPDZ3 was required for the interaction between KIF5A and PSD-95 (Fig. 4), we examined whether ADPDZ3 expression affects the dendritic level of PSD-95. The cultured hippocampal neurons were infected with Sindbis viruses encoding either GFP or GFP-ADPDZ3 and subjected to immunostaining using monoclonal anti-PSD-95 antibody or polyclonal anti-synapsin I antibody. The monoclonal PSD-95 antibody did not detect overexpressed ADPDZ3 particles (data not shown). ADPDZ3 domain expression significantly reduced the number of PSD-95 particles, but not synapsin I particles (Fig. 5a−c), suggesting that inhibition of the interaction between PSD-95 and KIF5A blocks PSD-95 dendritic localization. This reduction was more dramatic in distal dendritic regions than in proximal regions (Additional file 2: Figure S2). Accordingly, ADPDZ3 expression resulted in a significant reduction of synapse number, possibly due to the reduced dendritic transport of PSD-95 (Fig. 5a, d).

### ADPDZ3 expression reduces surface GluA1 level in dendrites

**Fig. 6:**
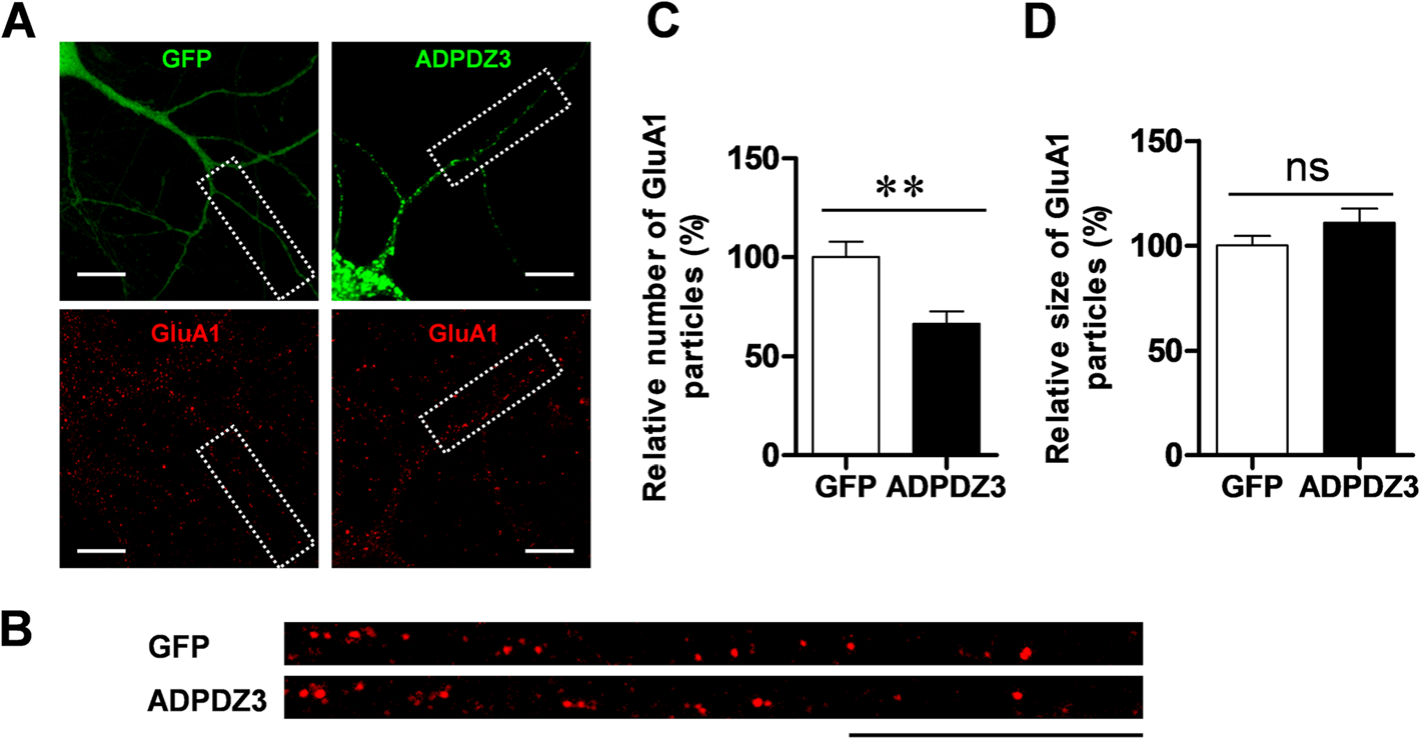
ADPDZ3 expression reduces surface glutamate receptor 1 (GluA1) in dendrites. Cultured rat hippocampal neurons were infected with Sindbis viruses encoding GFP or GFP-ADPDZ3 and incubated for 9 h to allow expression. The cultures were then subjected to immunostaining using polyclonal GluA1 antibody and Cy3-conjugated anti-mouse IgG antibody. They were visualized using confocal microscopy. **a**, **b** Representative images of expressed neurons and selected dendrites in the analyses. Dotted boxes indicate analyzed dendrites. Scale bar: 20 μm. **c**, **d** ADPDZ3 expression reduced the number of surface GluA1 particles (GFP: 100.00% ± 7.83%, *n* = 18, 1486 μm; ADPDZ3: 66.50% ± 6.21%, *n* = 14, 1254 μm; ** *P* < 0.01), but not size of the surface GluA1 particles (GFP: 100.00% ± 4.51%, n = 18; ADPDZ3: 110.80% ± 6.82%, n = 14, ns: not significant). N values indicate number of dendrites from multiple neurons derived from three different batches

Since expression of the ADPDZ3 domain reduced the level of PSD-95—a major scaffolding protein in the synapses of excitatory neurons [1, 2, 4, 29], it is possible that expression of AMPA receptor at postsynaptic membranes is also affected. To test this idea, we examined whether ADPDZ3 expression also reduces the level of surface AMPA receptors. After ADPDZ3 expression, surface GluA1 was immunostained and evaluated by imaging analysis. Consistent with the results of our PSD-95 assay, ADPDZ3 expression significantly reduced the number of surface GluA1 particles (Fig. 6), supporting the idea that expression of AMPA receptor at synaptic membranes is dependent on KIF5A-mediated transport of PSD-95 to synaptic regions.

### Staufen modulates the association between PSD-95 and KIF5A

**Fig. 7:**
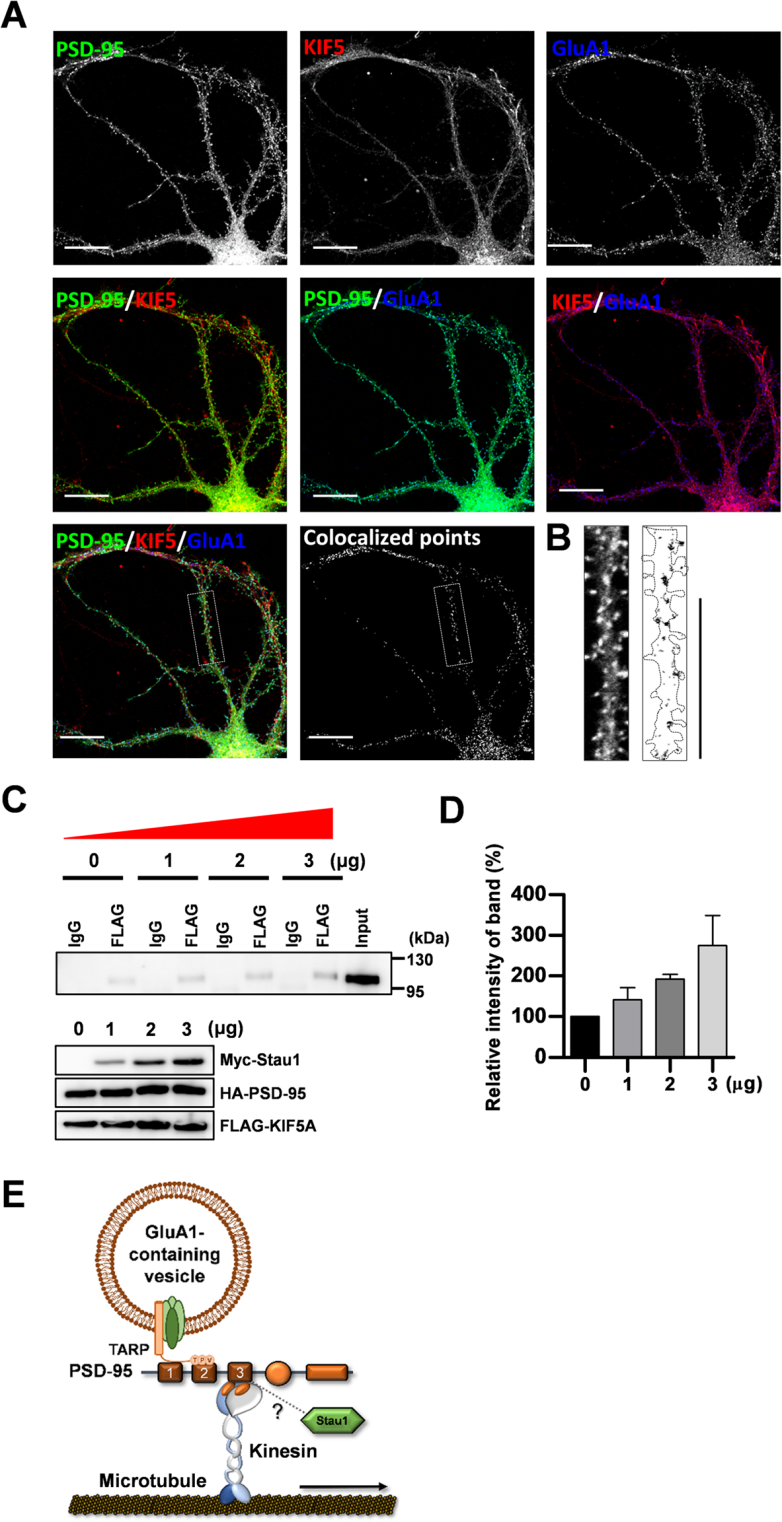
Complexes of PSD-95-KIF5 colocalized with GluA1 particles in dendrites. Cultured hippocampal neurons were transfected with PSD-95-GFP constructs and incubated for days. The cultures were immunostained with monoclonal anti-PSD-95 antibody and polyclonal GluA1 antibody; they were subsequently stained with C3-conjugated anti-mouse IgG and Alexa−Fluor® 647 anti-rabbit IgG antibody. **a** Representative images of immunostaining in the first row. Each image was merged to show colocalization in the second row. A colocalized image of PSD-95 and KIF5 was collated with the image of GluA1. The colocalized points appeared white. Scale bar: 20 μm. **b** Boxed dendrites were enlarged to see the colocalization of GluA1 with the complex of PSD-95-KIF5A. **c** Staufen expression increased the association of PSD-95 and KIF5A. HA-PSD-95 and FLAG- KIF5A were cotransfected with 1, 2, or 3 μg of Myc-Staufen, or without Myc-Staufen as a control. After immunoprecipitation using anti-FLAG antibody or mouse IgG, immunoprecipitates were analyzed by Western blotting using anti-HA antibody. The lower panel shows expression of each group. **d** Quantified data of Western blot analyses (0 μg of Staufen: 100.0% ± 0.00%, n = 3; 1 μg of Staufen: 141.6% ± 16.96%, n = 3; 2 μg of Staufen: 192.3% ± 6.59%; 3 μg of Staufen; 274.4% ± 42.30%, *n* = 3). N values indicate the number of independent experiments. **e** Schematic diagram showing GluA1-containing vesicle transport mediated by PSD-95-KIF5A complex in dendrites. TARP: transmembrane AMPA receptor regulatory protein

Previous studies demonstrated that AMPA receptor vesicles are transported to the dendrites by KIF5 through the interaction between glutamate receptor interacting protein 1 (GRIP1) and kinesin heavy chain [22, 28]. Thus, we investigated whether KIF5 − associated with PSD-95 interacts with AMPA receptor vesicles. GFP-tagged PSD-95 was expressed in the cultured hippocampal neurons and immunostained using anti-KIF5 and GluA1 antibodies to analyze colocalization among PSD-95, KIF5, and GluA1. As shown in Fig. 7a and b, we found colocalization among these three proteins in dendrites, suggesting that AMPA receptor vesicles are transported to the dendrites by PSD-95 − KIF5A complexes. Next, we explored to identify whether any other linker or adaptor proteins are involved in the interaction between KIF5 and PSD-95. Because a previous study showed that Staufen functions as a linker protein for RNA granules, which are known as a cargo of KIF5 [30], and synaptic localization of PSD-95 depends on the expression or availability of Staufen [31], we examined whether Staufen expression modulates the association between PSD-95 with KIF5A. We gradually increased an amount of Myc-tagged Staufen to the cells expressing FLAG-tagged KIF5A and HA-tagged PSD-95, and measured interactions between KIF5A and PSD-95 using immunoprecipitation. Our results showed that Staufen expression increased the interaction in dose-dependent manner (Fig. 7c, d), indicating that Staufen functions as a linker or adaptor between cargoes and KIF5.

## Discussion

In the present study, we provide evidence that KIF5 (kinesin-1 family), also known as conventional kinesin, is a motor protein for PSD-95 dendritic transport. A previous study [15] reported that KIF1Bα (kinesin-3 family) associates with the C-terminal regions of PSD-95, while another [23] reported KIF13B (GAKIN) interacted with PSD-95 in epithelial cells. Lin et al. (2012) showed that KIF3A (kinesin-2 family) is involved in GluA2 trafficking in combination with GRIP 1 and PSD-95 in retina cells [32]. In the line with this idea, our results showed that inhibiting KIF5A by expressing a dominant-negative form (ΔMD) did not completely block PSD-95 localization (Fig. 1). In addition to KIF5, we also identified interaction of PSD-95 with KIF3A (data not presented). These results indicate that multiple motor proteins are involved in PSD-95 transport in various neuron types. Despite their somewhat overlapping expression level, each KIF displays a distinct expression pattern in the brain [33]. KIF5A is highly enriched in neurons of the cortex and the hippocampus [17, 25, 33], KIF1B in motor neurons in the medulla oblongata and the spinal cord [34], and KIF3A in granular cells of the cerebellum [35], while KIF13A in non-nerve tissues [1, 24]. It is thus possible that dendritic transport of scaffolding proteins such as PSD-95 is regulated by distinct KIF isoforms in different brain areas.

Many previous studies have indicated that PSD-95 work as an adaptor between a motor protein and receptor−containing vesicle cargoes. For example, Mint1/X11 (mLin-10)—a PDZ domain−containing protein—interlinks NMDA receptor−containing vesicles and KIF17, which is a member of kinesin-2 family [21]. Glutamate [NMDA] receptor subunit 1 (GluN1) and GluN2 − containing vesicles are transported to dendrites with SAP97 and calcium/calmodulin-dependent serine protein kinase (CASK), while PDZ domain−containing MAGUKs are transported by KIF17 [36]. In addition, by directly interacting with GRIP1, which is another PDZ domain−containing scaffolding protein, KIF5C is reported to transport GluA2 − containing vesicles to dendrites [28]. Thus, GRIP1 interlinks *N*-cadherin and GluA2-containing vesicles, transporting them into the dendrites [22]. Interestingly, Huntingtin−associated protein 1 (HAP 1) works as an adaptor molecule for the dendritic transport of GABA_A_ receptor-containing vesicles in the inhibitory synapses [37]. Scaffolding and motor proteins carry out dendritic transport and synaptic localization of transmembrane proteins, such as receptors [38]. Our data agree with these previous results, because GluA1 expression on dendritic membranes was dependent on KIF5 − mediated transport of PSD-95 (Figs. 6, 7a).

GRIP 1 directly interacts with KIF5 through PDZ6 and PDZ7 domain, functioning as an adaptor between KIF5A and GluA2-containing vesicles [28]. The protein mLin-10 also directly interacts with KIF17 via the PDZ1 domain [21]. It is likely that PSD-95 directly interacts with KIF5A, although we did not examine this possibility in the study. However, a previous study identified a putative PDZ interaction motif (class I: Ser/Thr − X − Val, S/TXV) [39] in the tail regions of KIF5A, and this PDZ interaction motif was only found in the KIF5A isoform [17]. The present study corroborated previous data [21, 28], showing that PSD-95 binding to KIF5 may steer KIF5A to dendrites, as occurs when an adaptor binds to a motor protein. Another previous study suggested that PSD-95 is associated with Staufen in synaptic regions [31]. Staufen also works as an adaptor protein for KIF5 cargoes [30]. Consistent with these studies, in the present study, Staufen expression increased the association of PSD-95 with KIF5 (Fig. 7c and d), indicating that Staufen might modulate the PSD-95-KIF5 complex. Further studies should investigate the detailed molecular configuration of scaffolding protein−motor protein transport complexes modulated by Staufen.

Motor protein expression increases the levels of the corresponding cargo protein or associated protein in dendrites [22]. In the present experiment, even though the dominant mutant form of KIF5A (ΔMD) significantly decreased the level of PSD-95 in dendrites, KIF5A (WT) did not increase PSD-95 transport (Fig. 1). Considering for multiple motors to be involved in the transport of PSD-95, the role of single protein might not be considerable. Alternatively, neuronal activity may be required to increase motor activity. Indeed, increases in expression levels of cargo proteins require neuronal activity [26]. The present study has suggested a new mechanism in the dendritic transport of PSD-95 and receptor-containing vesicles in glutamatergic synapses (Fig. 7e).

## Methods

### Cell cultures and transfections

Rat hippocampal neurons were isolated from 1-day-old pups (Sprague Dawely, Samtako, Osan, Republic of Korea). Cultures were maintained in Neurobasal–A (Life Technologies, Carlsbad, CA, USA) supplemented with B27 supplement (Life Technologies) at 37 °C and in 5% CO_2,_ as previously described [31]. The cultures were performed in accordance with the approved animal protocols and the guidelines of the Institutional Animal Care and Use Committee of Chungbuk National University (CBNUA-1049-17-01). Human embryonic kidney (HEK) 293 T cells were maintained in Dulbecco’s Modified Eagle’s Medium (Life Technologies) supplemented with 10% fetal bovine serum (Biowest, Nuaillé, France). With regards to transfection of the neurons, target genes were subcloned to a pSinRep5 vector for Sindbis viral expressions and packaged into Sindbis virion particles according to Invitrogen’s user manual (Invitrogen, Carlsbad, CA, USA). The Sindibis virion was directly added to the cultured neurons and incubated for 6–12 h. Transfection of the HEK cells was carried out by transferring DNA constructs using a calcium phosphate method.

### DNA constructs

Complete cDNA of mouse PSD-95 (disks large homolog 4, NM_007864) were amplified using PCR (mPSD95-R1-S → mPSD95-Xho-A) and inserted at the *EcoR*I/*Xho*I site of the pCS4-3xHA vector. To construct the mutant form of the PSD-95, full length or partial PSD-95 fragments were amplified using PCR and inserted at the *EcoR*I/*Xho*I site of the pCS4-3xHA vector (full length: mPSD95-R1-S → mPSD95-Xho-A; PDZ1: mPSD95-R1-S → PZD1-Xho-A; PDZ1–2: mPSD95-R1-S → PZD2-Xho-A; PDZ1–3: mPSD95-R1-S → PZD3-Xho-A; ∆GK: mPSD95-R1-S → SH3-Xho-A; GK: GMPK-R1-S → mPSD95-Xho-A). To allow fine mapping, PSD-95 fragments were amplified using PCR (ADPDZ3: mPSD95-ADPDZ3-R1-S → PDZ3-Xho-A2; PDZ3: mPSD95-PDZ3-R1-S → PDZ3-Xho-A2; AD: mPSD95-ADPDZ3-R1-S2 → AD-Xho-A). In the case of ADPD3 and PDZ3, the fragments were inserted at the *EcoR*I/*Xho*I site of the pCS4-3xHA vector. While in the case of AD, they were inserted at the *EcoR*I/*Xho*I site of the pCMV-myc vector. To allow Sindbis viral expression, GFP-tagged PSD-95 (PSD-95-GFP) and GFP-tagged ADPDZ3 (GFP-PDZ3) were amplified using PCR (PSD-95-GFP: mPSD95-Mlu-S → GFP-Sph-A; GFP-ADPDZ3: GFP-Mlu-S → PDZ3-Sph-A) and inserted at the *Mlu*I/*Sph*I site of the pSinRep5 vector.

The complete cDNA of mouse *Kif5a* (NM_008447) was amplified using PCR (mKIF5A-Bam-S → mKIF5A-Apa-A) and inserted at the *BamH*I/*Apa*I site of the pCMV-tag2B vector. To construct mutants, the KIF5A fragments were amplified using PCR and inserted at the *BamH*I/*Apa*I site of the pCMV-tag2B vector (full length: mKIF5A-Bam-S → mKIF5A-Apa-A; 636: mKIF5A-Bam-S → mKIF5A-636-Apa-A; 826: mKIF5A-Bam-S → mKIF5A-826-Apa-A; 906: mKIF5A-Bam-S → mKIF5A-906-Apa-A; ∆MD: mKIF5A-330-Bam-S → mKIF5A-Apa-A). To allow Sindbis viral expression, wild type and ∆MD mutant cDNA was amplified using PCR (mKIF5A-Sph-S → mKIF5A-Apa-A; ∆MD: mKIF5A-330-Sph-S → mKIF5A-Apa-A) and inserted at the *Sph*I/*Apa*I site of pSinRep5-mRFP, yielding the DNA constructs encoding for mRFP-tagged WT KIF5A or mRFP-tagged ∆MD. The complete cDNA of mouse *Kif5b* (NM_008448) was amplified using PCR (mKIF5B-Bam-S → mKIF5B-Apa-A) and inserted at the *BamH*I/*Apa*I site of the pCMV-tag2B vector. The complete cDNA of human *Kif5c* (NM_004522) was amplified using PCR (hKIF5C-R1-S → hKIF5C-Sal-A) and inserted at the *EcoR*I/*Sal*I site of the pCMV-tag2B vector. The cDNA clones of KIF5s were provided by Dr. EY Shin (Chungbuk National University). All PCR primers for PCR were purchased from Bioneer (Daejeon, Republic of Korea). Restriction enzymes used in our experiments were purchased from New England Biolabs (NEB, Ipswich, MS, USA).

mPSD95-R1-S: 5′- ggaattcaatggactgtctctgtatagtg-3′,

mPSD95-Xho-A: 5′-ccgctcgagtcagagtctctctcgggctg-3′

PDZ1-Xho-A: 5′-ccgctcgagtcacttctcagctgggggttt-3′

PDZ2-Xho-A: 5′-ccgctcgagtcaggccacctttaggtacac-3′

PDZ3-Xho-A: 5′-ccgctcgagtcaccgcttggggttgcttcg-3′

SH3-Xho-A: 5′-ccgctcgagtcagcgagcgtagtgcacttc-3′

GMPK-R1-S: 5′-ggaattcacccatcatcatccttggg-3′

mPSD95-ADPDZ3-R1-S: 5′-ggaattcaaagcccagcaatgcctacc-3′

PDZ3-Xho-A2: 5′-ccgctcgagtcagatgatcgtgaccgtctg-3′

mPSD95-PDZ3-R1-S: 5′-ggaattcaaggcggatcgtgatccatc-3′

AD-Xho-A: 5′-ccgctcgagtcaccttggttcccggggaa-3′

mPSD95-Mlu-S: 5′-cgacgcgtatggactgtctctgtatagtg-3′

GFP-Sph-A: acatgcatgcttacttgtacagctcgtcca-3′

GFP-Mlu-S: 5′-cgacgcgtgtcgccaccatggtgagc-3′

PDZ3-Sph-A: 5′-acatgcatgctcagatgatcgtgaccgtctg-3′

mKIF5A-Bam-S: 5′-cgggatccatggcggagactaacaac-3′

mKIF5A-Apa-A: 5′: 5′-tgggcccccttagctggctgctgtctc-3′

mKIF5A-636-Apa-A: 5′-tgggggcccttaatgctgtgagatgagcag-3′

mKIF5A-826-Apa-A: 5′-tgggggcccttaggaatgaatccccccac-3′

mKIF5A-906-Apa-A: 5′-tgggggcccttagtaccgcacggcttcttt-3′

mKIF5A-330-Bam-S: 5′-cgggatccgcctcagtgaatctggag-3′

mKIF5A-Sph-S: 5′-acatgcatgctcgaccaccatggcgga-3′

mKIF5A-330-Sph-S: 5′-acatgcatgcgcctcagtgaatctggag-3′

mKIF5B-Bam-S: 5′-cgggatccatggcggacccggcggag-3′

mKIF5B-Apa-A: 5′-agggggcccttacgactgcttgcctccac-3′

hKIF5C-R1-S: 5′-ggaattctatggcggatccagccgaa-3′

hKIF5C-Sal-A: 5′-cgacgtcgacttatttctggtagtgagtgg-3′

### Co-immunoprecipitation

For co-immunoprecipitation (co-IP), cell lysates were prepared by adding lysis buffer (150 mM NaCl, 1% IGEPAL® CA-630, 50 mM Tris·Cl; pH 8.0) supplemented with a protease inhibitor cocktail (Roche, Basel, Switzerland). The lysate was immunoprecipitated using 2–3 μg of antibody (specificity indicated in the figures), mouse immunoglobulin G (IgG; Sigma-Aldrich, St. Louis, MO, USA), and incubated with 50 μL of Protein G-Sepharose (GE Healthcare, Chicago, IL, USA). The immunoprecipitates were washed three times in 1 mL of ice-cold lysis buffer, followed by additional wash an additional time with 1 mL of 50 mM Tris·Cl (pH 8.0). The precipitated proteins were separated using sodium dodecyl sulfate-polyacrylamide gel electrophoresis (SDS-PAGE) (8%–12%). For western blot analysis, the blots were incubated using the antibody indicated in the figures. All co-IPs and western blot analyses were performed more than twice to confirm that the data were reproducible. The following antibodies were used in the co-IPs and western blot analyses: monoclonal anti-FLAG antibody (1:2000, Clone M2; Sigma-Aldrich), monoclonal anti-HA antibody (1:2000, Clone HA-7; Sigma-Aldrich), and monoclonal anti-Myc antibody (1:2000, Clone 9E10; Sigma-Aldrich).

### Immunocytochemistry and proximity ligation assay

For the immunocytochemistry, cultures were fixed using a fixative (4% paraformaldehyde, 4% sucrose, pH 7.2) and permeabilized using PBT (0.1% TritonX-100, 0.1% BSA in PBS). In the case of surface GluA1 immunocytochemistry, no permeabilization step was performed. The cultures were pretreated using the preblock solution (2% BSA, 0.08 TritonX-100 in PBS) for 1 h and each antibody was directly added to the preblock solution for 2 h. The following antibodies were used for staining, each at a dilution of 1:50; monoclonal anti-PSD-95 antibody (clone 6G6-1C9; Affinity Bioreagents, Golden, CO, USA), polyclonal anti-PSD-95 antibody (Cell Signaling, Danvers, MA, USA), monoclonal anti-kinesin antibody (Clone: H2; Millipore, Temecula, CA, USA), polyclonal anti-synapsin I antibody (Millipore), polyclonal anti-GluA1 antibody (Upstate, Lake Placid, NY), polyclonal anti-GluA1 antibody (Alomone Labs, Jerusalem, Israel) for surface GluA1.The following antibodies were used for secondary staining, each at a dilution of 1:200: Alexa Fluor® 488 anti-rabbit IgG antibody (Molecular Probes, Eugene, OR, USA), Cy3-conjugated anti-mouse IgG antibody (Jackson ImmunoResearch Laboratories, West Grove, PA, USA), Cy3-conjugated anti-rabbit IgG antibody (Jackson ImmunoResearch Laboratories), and Alexa Fluor® 647 anti-rabbit IgG antibody (Molecular Probes).

For PLA using Duolink® In Situ-Fluorescence (Sigma-Aldrich), the cultures were infected with Sindbis viruses encoding GFP to visualize whole dendritic structures and then fixed as described above; rabbit polyclonal anti-PSD-95 antibodies (Cell Signaling) and mouse monoclonal anti-KIF5 antibodies (Clone H2, Millipore) were then used. All procedures were performed according to the manufacturers’ instructions. The nucleus of each neuron was stained with 4′,6-diamidino-2-phenylindole (DAPI, Sigma-Aldrich). Immunostaining and PLA were visualized using confocal microscopy (Zeiss 710; Carl Zeiss, Oberkochen, Germany).

### Image analysis

Secondary or tertiary dendrites with a similar diameter were selected from acquired neuron images and straightened using a plugin of ImageJ program (ver 1.47; National Institute of Health, Bethesda, VA, USA). The images of straightened dendrites were transited to threshold images. The number and size of PSD-95 or GluA1 particles were measured using the particle analysis plugin. Colocalization was measured from the threshold images using colocalization plugins and represented using either Pearson’s correlation coefficient (R − value) or a percentage. All image analyses were performed by blind experiment.

### Statistical analysis

Normality of the data was assessed using either the Kolmogorov-Smirnov test or the D’Agostino and Pearson omnibus normality tests. If the data followed Gaussian distribution, a Student’s *t*-test was performed to determine statistical significance between two groups, while analysis of variance (ANOVA) was performed among three or more groups, with Newman Keul’s analysis used as a post hoc analysis. If the data did not follow Gaussian distributions, the non-parametric Mann-Whitney test was performed to determine statistical significance between two groups, while the Kruskal-Wallis test combined with Dunn’s multiple comparison test was performed among three or more groups. All statistical analyses were performed using GraphPad prism (ver 5.02; GraphPad Software, San Diego, CA, USA).

## Supplementary information

**Additional file 1: Figure S1.** All isoforms of KIF5 interact with PSD-95. Cultured HEK cells were transfected with plasmids of HA-tagged PSD-95 and FLAG-tagged KIF5A or FLAG-tagged KIF5B, or FLAG-tagged KIF5C. The lysates were used for IP using monoclonal anti-FLAG antibody (2 μg, Clone M2, Sigma-Aldrich) and then the precipitates were analyzed by Western blotting assay using monoclonal anti-HA antibody (1:2000, Clone HA-7; Sigma-Aldrich). The bottom blots show expression of each protein used in immunoprecipitations.

**Additional file 2: Figure S2.** ADPDZ3 expression reduces the level of PSD-95 in dendrites. Cultured rat hippocampal neurons were infected with Sindbis viruses encoding GFP or GFP-ADPDZ3 and incubated for 9 h to allow expression. The cultures were then subjected to immunostaining using monoclonal anti-PSD-antibody and Cy3-conjugated anti-mouse IgG antibody. They were visualized using confocal microscopy. (A) Representative images of expressed neurons. Arrows indicate analyzed dendrites. Scale bar: 20 μm. (B) ADPDZ3 expression reduced the number of PSD-95 particles per 10 μm of dendrites (GFP: 5.41 ± 0.24, *n* = 14, 1188 μm; ADPDZ3: 4.61 ± 0.26, *n* = 16, 1542 μm). (C) ADPDZ3 expression did not change the size of PSD-95 particles (GFP: 0.34 μm^2^ ± 0.01 μm^2^, n = 14, 1188 μm; ADPDZ3: 0.32 μm^2^ ± 0.01 μm^2^, n = 16, 1542 μm). (D) Representative images of dendrites divided into constant length (25 μm). (D) Relative number of PSD-95 particles were reduced in the distal regions (1–25 μm, GFP: 100.00% ± 0.00%, *n* = 13, ADPDZ3: 100.00% ± 0.00%, *n* = 15; 26–50 μm, GFP: 92.71% ± 5.93%, n = 13, ADPDZ3: 86.32% ± 6.70%, n = 15; 51–75 μm, GFP: 76.73% ± 10.34%, n = 13, ADPDZ3: 68.66% ± 5.65%, n = 15; 76–100 μm, GFP: 71.60% ± 7.88%, *n* = 12, ADPDZ3: 48.60% ± 2.71%, n = 14). (F) Relative size of PSD-95 particles was not changed in all dendritic regions (1–25 μm, GFP: 100.00% ± 0.00%, n = 13, ADPDZ3: 100.00% ± 0.00%, n = 15; 26–50 μm, GFP: 98.26% ± 5.83%, n = 13, ADPDZ3: 111.98% ± 7.62%, n = 15; 51–75 μm, GFP: 100.87% ± 7.50%, n = 13, ADPDZ3: 108.83% ± 6.05%, n = 15; 76–100 μm, GFP: 109.74% ± 9.18%, n = 12, ADPDZ3: 120.50% ± 11.38%, n = 14). N values indicate n dendrites from n neurons.

## Data Availability

All data generated or analyzed during this study are included in this published article and its supplementary information files.

## Abbreviations

AD: Association domain
AMPA: α-Amino-3-hydroxy-5-methyl-4-isoxazolepropionic acid
ANOVA: Analysis of variance
CASK: Calcium/calmodulin-dependent serine protein kinase
DAPI: 4′,6-Diamidino-2-phenylindole
DLG: Discs large homology
GABA: γ-Aminobutyric acid
GFP: Green fluorescent protein
GluA1: Glutamate receptor 1
GRIP1: Glutamate receptor interacting protein 1
HA: Hemagglutinin
HEK: Human embryonic kidney
IgG: Immunoglobulin G
KIF: Kinesin superfamily protein
MAGI-2: Membrane associated guanylate kinase inverted-2
MAGUK: Member of membrane-associated guanylate kinase
NMDA: *N*-methyl-D-aspartate
PBS: Phosphate buffered saline
PLA: Proximity ligation assay
PSD-95: Postsynaptic density protein 95
SAP97: Synapse-associated protein-97
S-SCAM: Synaptic scaffolding molecule
TARP: Transmembrane AMPA receptor regulatory protein
TrkB: Tyrosine receptor kinase B
ZO-1: Zona occludens-1
