# Supplementary figures and images for: Postsynaptic density protein 95 (PSD-95) is transported by KIF5 to dendritic regions
Source: Mol Brain. 2019 Nov 21;12:97. doi: 10.1186/s13041-019-0520-x (PMC6873588; doi:10.1186/s13041-019-0520-x)

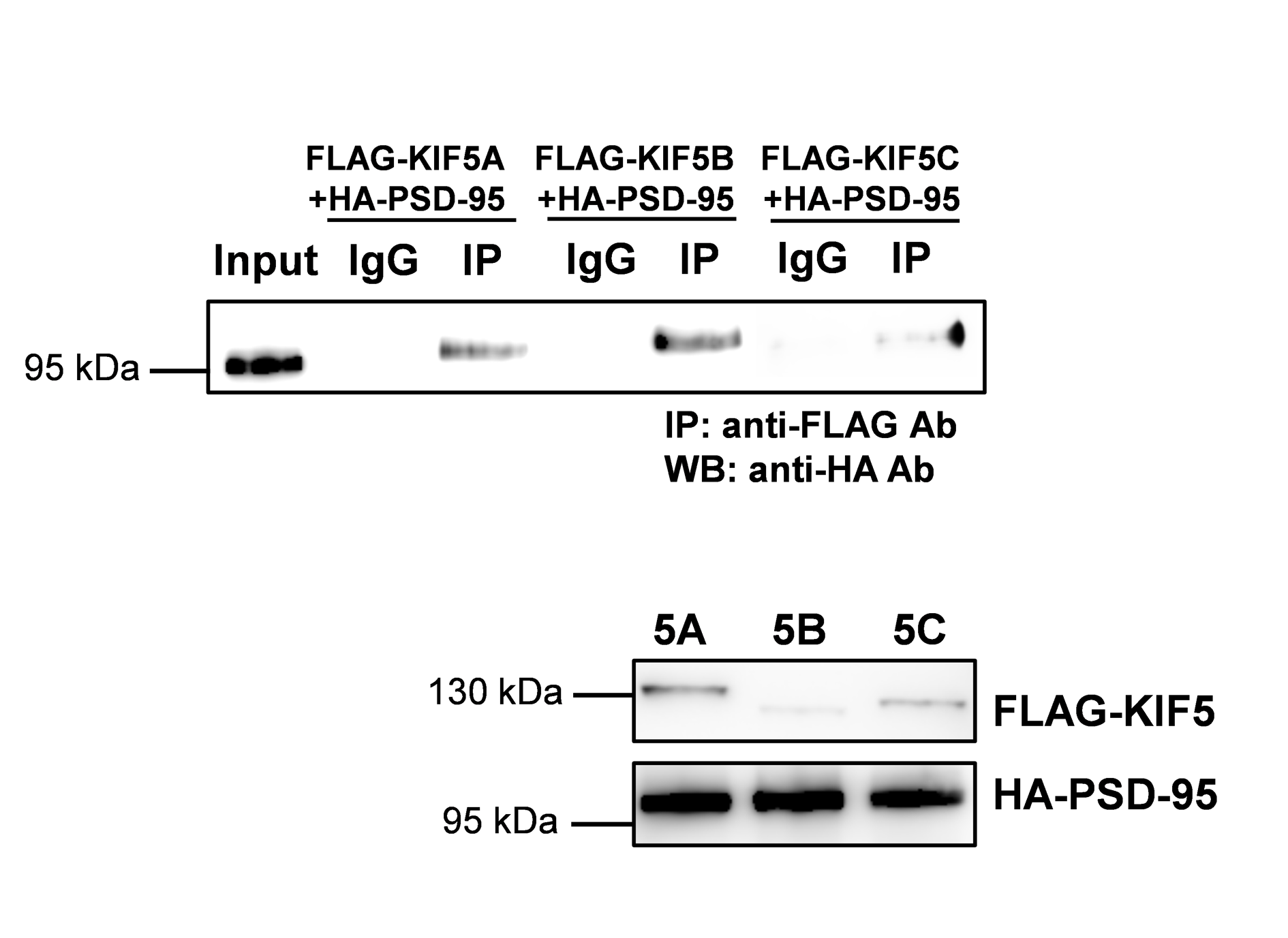

Supplement: Supplementary file 1 — Additional file 1: Figure S1. All isoforms of KIF5 interact with PSD-95. Cultured HEK cells were transfected with plasmids of HA-tagged PSD-95 and FLAG-tagged KIF5A or FLAG-tagged KIF5B, or FLAG-tagged KIF5C. The lysates were used for IP using monoclonal anti-FLAG antibody (2 μg, Clone M2, Sigma-Aldrich) and then the precipitates were analyzed by Western blotting assay using monoclonal anti-HA antibody (1:2000, Clone HA-7; Sigma-Aldrich). The bottom blots show expression of each protein used in immunoprecipitations. [file 13041_2019_520_MOESM1_ESM.tif]

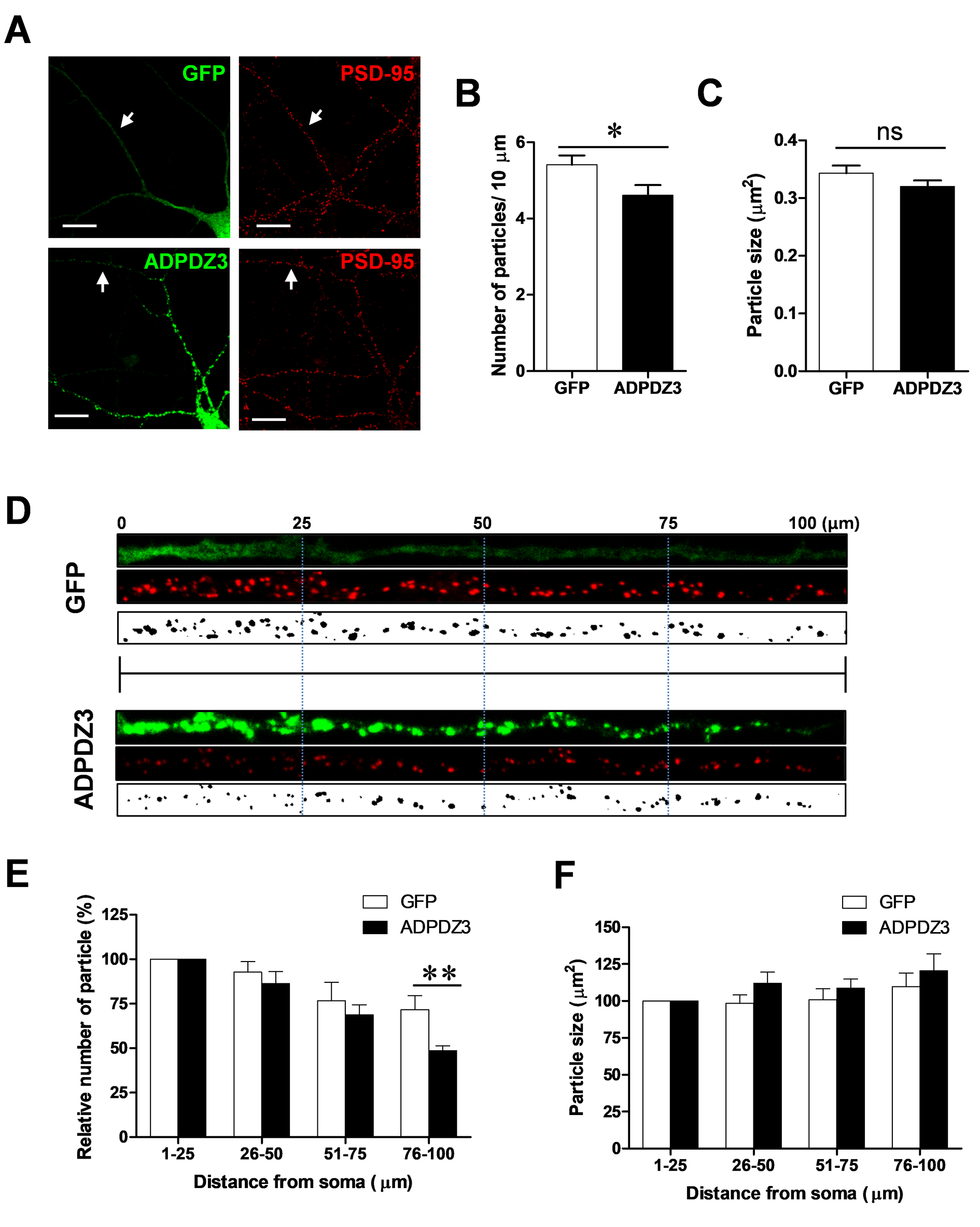

Supplement: Supplementary file 2 — Additional file 2: Figure S2. ADPDZ3 expression reduces the level of PSD-95 in dendrites. Cultured rat hippocampal neurons were infected with Sindbis viruses encoding GFP or GFP-ADPDZ3 and incubated for 9 h to allow expression. The cultures were then subjected to immunostaining using monoclonal anti-PSD-antibody and Cy3-conjugated anti-mouse IgG antibody. They were visualized using confocal microscopy. (A) Representative images of expressed neurons. Arrows indicate analyzed dendrites. Scale bar: 20 μm. (B) ADPDZ3 expression reduced the number of PSD-95 particles per 10 μm of dendrites (GFP: 5.41 ± 0.24, n = 14, 1188 μm; ADPDZ3: 4.61 ± 0.26, n = 16, 1542 μm). (C) ADPDZ3 expression did not change the size of PSD-95 particles (GFP: 0.34 μm2 ± 0.01 μm2, n = 14, 1188 μm; ADPDZ3: 0.32 μm2 ± 0.01 μm2, n = 16, 1542 μm). (D) Representative images of dendrites divided into constant length (25 μm). (D) Relative number of PSD-95 particles were reduced in the distal regions (1–25 μm, GFP: 100.00% ± 0.00%, n = 13, ADPDZ3: 100.00% ± 0.00%, n = 15; 26–50 μm, GFP: 92.71% ± 5.93%, n = 13, ADPDZ3: 86.32% ± 6.70%, n = 15; 51–75 μm, GFP: 76.73% ± 10.34%, n = 13, ADPDZ3: 68.66% ± 5.65%, n = 15; 76–100 μm, GFP: 71.60% ± 7.88%, n = 12, ADPDZ3: 48.60% ± 2.71%, n = 14). (F) Relative size of PSD-95 particles was not changed in all dendritic regions (1–25 μm, GFP: 100.00% ± 0.00%, n = 13, ADPDZ3: 100.00% ± 0.00%, n = 15; 26–50 μm, GFP: 98.26% ± 5.83%, n = 13, ADPDZ3: 111.98% ± 7.62%, n = 15; 51–75 μm, GFP: 100.87% ± 7.50%, n = 13, ADPDZ3: 108.83% ± 6.05%, n = 15; 76–100 μm, GFP: 109.74% ± 9.18%, n = 12, ADPDZ3: 120.50% ± 11.38%, n = 14). N values indicate n dendrites from n neurons. [file 13041_2019_520_MOESM2_ESM.tif]
